# The 8^th^ Annual Bio-Ontologies Meeting

**DOI:** 10.1002/cfg.499

**Published:** 2005

**Authors:** Robert Stevens, Robin McEntire, Phillip Lord, James. A. Butler

**Affiliations:** 1 School of Computer Science University of Manchester Oxford Road Manchester M13 9PL UK; 2 GlaxoSmithKline Informatics & Knowledge Management 709 Swedeland Road King of Prussia PA 19406 USA


**Comparative and Functional Genomics**
*Comp Funct Genom* 2005; **6**: 370–372.
Published online in Wiley InterScience (www.interscience.wiley.com). **DOI:** 10.1002/cfg.499

**Editorial**

### The 8th Annual Bio-Ontologies Meeting

Robert Stevens[1]*, Robin McEntire[2], Phillip Lord[1], and James. A. Butler[2]
[1]*School of Computer Science, University of Manchester, Oxford Road, Manchester, United Kingdom, M13 9PL*
[2]*GlaxoSmithKline, Informatics & Knowledge Management 709 Swedeland Road, King of Prussia, PA 19406, USA*

**Correspondence to:*
*Dr. Robert Stevens, School of Computer Science, University of Manchester, Oxford Road, Manchester, United Kingdom, M13 9PL.*
*E-mail:*
*robert.stevens@manchester.ac.uk*

Received: 20 November 2005
Accepted: 22 November 2005

### Introduction

The eighth annual Bio-Ontologies meeting was held on 24th June at ISMB 2005 in Detroit. Its principle aim over the years has been to build a community forum that brings together biologists and computer scientists to discuss the development and use of ontologies within the domain of biology. During this time, it has aimed to stimulate discussion about the role of Ontologies and their associated technologies for describing, sharing, analysing and searching knowledge about biological systems.

Ontology papers have been appearing in the ISMB proceedings since the conference's inception. Pioneering work by Peter Karp with EcoCyc [1] demonstrated the use of ontologies as the backbone of knowledge bases. Since the first Bio-Ontologies meeting in 1998, ontologies have an increasing presence in bioinformatics; particularly with the advent of the Gene Ontology [2] that demonstrated the value of supplementing genomic annotation with ontology terms. This meeting, like its predecessors from 1998 onwards, has been dominated by talks about the Gene Ontology.

This interest in ontology from the biomedical sciences is intersecting with interest in bio-ontologies and their use from the outside world in the form of the Web community; the Semantic Web vision now being actively promoted as the "next generation" Web makes heavy use of ontological technology; this, in turn, is leading to increasing provision of mature tools and experience, lessening the activation energy for those biologists who wish to develop or use ontologies. In the main ISMB conference, the use of semantic web, or at least its technologies, was a recurrent theme.

A recurrent issue in this year's meeting, highlighted particularly by Mark Musen's keynote, are the attempts to move bio-ontology development to a more industrial scale (Further information about this talk and others mentioned in this article are available from the Bio-Ontologies Website). Currently, ontology development is often carried out by small groups of individuals, mirroring much of biology before the advent of community wide sequencing efforts. With the large numbers of ontologies currently being produced (see **http://obo.sourceforge.net**, for examples), orthogonality, maintenance and consistency are all becoming key issues. At the current time, there is a relatively poor understanding of both the best practises which are necessary and the technology which will be required to support these best practices; what, for instance, are the best mechanisms for peer review of ontologies; is

Copyright © 2006 John Wiley & Sons, Ltd.

centralised management necessary as has happened with human gene naming, or are more "free-for-all", decentralised approaches possible? This theme was re-visited in the day's panel session.

### Panel session

This year we had a post-lunch panel session with the following panelists:

- Mark Musen, Stanford University;
- Larry Hunter, University of Colorado;
- Judith Blake, Jackson Lab;
- Eric Neumann, W3C co-chair, Health-care and Life Sciences Interest Group.

In the current context of rapidly growing activity in the bio-ontology sector and possible new horizons in the Semantic Web, there are many contentious issues facing our community. With this in mind, we gave the panelists the following questions with which to focus discussion:

1. We should be more authoritarian and less liberal in the building of Bio-Medical Ontologies.

### Background:

As the development of bio-medical ontologies has become more widespread, the development of multiple ontologies with overlapping terms is inevitable (and is, to some extent, already happening).

Currently, a very "free market" approach is being followed. Is this a strength? Or should it be replaced with something more centralised, similar to, for example, the Human Gene Nomenclature Committee.

2. We are better at developing Bio-Medical Ontologies than we are at using them.

### Background:

Bio-Medical ontologies now have a broad spread over the subject area. The main use of these ontologies is to annotate records which we then retrieve by query or navigation. Should we be doing more with these ontologies? Even this narrow use lacks good user facing and reusable tooling.

3. The future for Bio-Medical ontologies is to be the semantic infrastructure for the computationally enabled systems biology view of life.

### Background:

In the Semantic Web vision, data and services are described semantically, such that they become computationally amenable. Bioinformatics is in a strong position to realise this vision, which might affect both the way the world uses data, and the way biologists view life. Is this either possible, or desirable, or both?

In the 90 minutes for the panel session, we only managed to cover the first question. In all likelihood, this is due to the pertinence of the question. As a community, we now have a great number of ontologies available. As ontologies are meant to enable a shared understanding of a domain, it would be good if we did not have conflicting ontologies, but did have a common style, etc. Should such an effort be policed? By and large, there was a consensus on the panel and in the audience–that some kind of monitoring was in order. Larry Hunter, in rejecting authoritarianism, invoked government by the people for the people. In the best libral tradition, it was felt that control is needed but openness and feedback from users was the best way to gain control. This discussion was particularly interesting in the light of the new National Center for Biomedical Ontology [3].

### Research talks

All of the abstracts for these talks and the posters may be found at **http://bio-ontologies.man.ac.uk.** Many of the research talks also touched on the techniques of the Semantic Web. Several people discussed applications of automated reasoning techniques: to enable complex querying over data from the yeast community (Baker *et al.*) or toward the automation of protein classification as part of the process of genome annotation (Wolstencroft *et al.*). Ontologies are increasingly being used statistically, often to increase understanding of experimental results–in the case of Vailaya *et al.* this was microarray data.

Broadening this theme a little, we also had a talk from Daniel McShan about using an ontology that described the features by which a member of a class of organic molecules, such as alcohols or amino

Copyright © 2006 John Wiley & Sons, Ltd.

acids, would be recognised. Such an ontology, when given a concrete chemical could classify that chemical. Olivier Bodenreider presented a paper by Francisco Azuaje *et al.* on the use of similarity measures working over ontologies as a method of assigning function.

One of the best aspects of the meeting is that every year the audience can find out about new ontologies and new developments in existing ontologies. One of these was a talk by Jun Liu about developing a provenance ontology for biological images. This complements other activities in this area, such as controlled vocabularies for evidence as seen for GO annotations [4]. Several more ontologies were presented in the poster session. Finally, Olivier Bodenrider presented his second talk on generating the implied relationships between the Gene Ontology and other ontologies, such as those for chemicals. This “enrichment” of an ontology formed a good link to the next series of talks about ontology development.

Several talks described new techniques for ontological engineering; new logics better able to describe changes in biological systems over time (Ramakrishnan *et al.*) or more formal treatment of pathological anatomical features (Smith *et al.*). This year, Bio-Ontologies was also able to hold a well attended poster session. Several of these described new warehousing environments (Dewey *et al.*; Karp *et al.*) or tools (Stephens *et al.*; Zheng *et al.*). Finally, a number of posters described existing projects from several view points (Cary and Luciano; Harris; Whetzel *et al.*).

### Future plans

One of the surprises about Bio-Ontologies 2005 was the increase in the number of paper submissions, to around 30. We were lucky to be able to accommodate much excellent work with the late introduction of a poster session. Given this, for next year, we hope to modify the publication process–we would like authors to have more space to explain their science than we have currently been able to provide.

We are currently investigating ways of interacting better with other SIGs. The programme at ISMB is now very full, with many different SIGs providing excellent science, but with conflicting schedules. For 2006, we are investigating co-ordinating with BioLink; the synergy between ontological representation and natural language techniques is a natural one. This should ensure that attendees can get maximal benefit from both programmes. We welcome any input via the bio-ont-sig@cs.man.ac.uk email

Originally, the main use of ontologies within biology was to enable a *de facto* integration between different data sources, by providing a common vocabulary. It is clear, now, that there are many uses beyond this. The use of ontologies is becoming common place in data analysis, model building and data validation. It is also clear from this years Bio-Ontologies meeting that bio-Medical scientists are no longer just using ontological technologies; they are contributing to their advance.

### Acknowledgements

We would like to thank GSK for their support for our invited speaker Professor Mark Musen.

### References

1. Keseler IM, Collado-Vides J, Gama-Castro S, *et al.* 2005. EcoCyc: a comprehensive database resource for Escherichia coli. *Nucl. Acids Res* **33**: D334–337.
2. The Gene Ontology Consortium. 2000. Gene Ontology: Tool for the Unification of Biology. *Nature Genetics* **25**: 25–29.
3. **http://www.bioontology.org/**.
4. **http://www.geneontology.org**.

Copyright © 2006 John Wiley & Sons, Ltd.
